# Investigation of biological activities of the flowers of *Lagerstroemia speciosa*, the Jarul flower of Bangladesh

**DOI:** 10.1186/s12906-018-2286-6

**Published:** 2018-08-06

**Authors:** Tasnuva Sharmin, Md. Shahidur Rahman, Habiba Mohammadi

**Affiliations:** 1 Department of Pharmaceutical Chemistry, Faculty of Pharmacy, University of Dhaka, Dhaka, Bangladesh; 2 Department of Chemistry, Faculty of Science, University of Dhaka, Dhaka, Bangladesh; 3 Department of Pharmacy, State University of Bangladesh, Dhaka, Bangladesh

**Keywords:** *Lagerstroemia speciosa*, Free radical scavenging activity, Brine shrimp lethality, Thrombolysis, Disc diffusion assay, Zone of inhibition, Writhing, Anti-nociception, Tail tipping method

## Abstract

**Background:**

*Lagerstroemia speciosa* (L.) Pers. (Family: Lythraceae) is used in traditional medicine in the treatment of diarrhea, diabetes and other diseases. The study was performed to conduct antioxidant, cytotoxic, thrombolytic, membrane stabilizing, antimicrobial, peripheral and central analgesic and hypoglycemic activity assays and phenobarbitone sodium–induced sleeping time test using crude methanol extract of flowers of *L. speciosa* and its different partitionates.

**Method:**

The antioxidant potential was evaluated by determining the ability of the samples to scavenge 1, 1-diphenyl-2-picrylhydrazyl (DPPH) free radical. The cytotoxic potential was examined following the procedures of brine shrimp lethality bioassay. Thrombolytic potential was assayed using *streptokinase* as standard. The samples were subjected to membrane stabilizing activity assay under heat induced condition. Antimicrobial potential was observed by disc diffusion method. The ability of the extract to inhibit writhing induced by acetic acid was determined in peripheral analgesic activity assay. The extract was also tested for central analgesic and hypoglycemic activities by tail flicking and tail tipping methods in Swiss albino mice model, respectively. CNS depressant activity was evaluated by an assay in which sleep was induced in mice using phenobarbitone sodium.

**Results:**

The chloroform soluble fraction of *L. speciosa* extract demonstrated the highest antioxidant activity (IC_50_ = 4.20 ± 0.41 μg/ml) while the most prominent cytotoxic potency was showed by hexane soluble fraction (LC_50_ = 2.00 ± 0.31 μg/ml). Among the test samples, the carbon tetrachloride soluble fraction induced clot lysis (64.80 ± 0.27%) and prevented heat induced haemolysis (41.90 ± 0.10%) to the maximum extent. The largest zone of inhibition (19.0 mm) against *Staphylococcus aureus,* was also observed for the same fraction. In peripheral analgesic activity assay, 16.68% inhibition of writhing was documented for the *L. speciosa* extract (400 mg/kg body weight dose). The extract (400 mg/kg dose) also reduced blood sugar level by 56.12% after three hours of administration of glucose solution. In CNS depressant activity assay, mice of the sample group slept for shorter period of time compared to control group.

**Conclusions:**

From our investigation, it can be suggested that, the extract should be further studied for possible phytochemicals responsible for the observed biological activities.

## Background

*Lagerstroemia speciosa* (L.) Pers. (Synonyms: *Adambea glabra* Lam., *Lagerstroemia major* Retz., *Munchausia speciosa*) is locally known as Jarul or Banaba (Family: Lythraceae). It is a small to medium sized or rarely large deciduous or semi-deciduous tree that grows abundantly in tropical and subtropical regions [1, 2]. Almost every plant part of *L. speciosa* is found to have several important biological properties. Leaves, roots and bark have several traditional uses [3, 4]. Leaf is used in hypercholesterolemia, hypertension, diabetes, different renal dysfunctions and to prepare slimming tea. Bark of the plant is locally used to treat diarrhea [4, 5]. The leaf extract of *L. speciosa* is reported as nephroprotective [6], hepatoprotective [7] and to have diuretic activity [8]. Leaf extract has been reported to have antioxidant activity while bark extract has been found to show cytotoxicity against *Artemia salina* [9–12]. Because of its anti-diabetic effects with no evident side effect, its use is considered the most efficacious and practical approach for 'ethno-pharmacologically' managing diabetes [13]. Herbal products such as Banabamin and Glucosol TM have been recently marketed from this ‘natural plant insulin’ [14, 15]. Its hypoglycemic effect has been evaluated in rabbit [16], mouse [17], alloxan-induced diabetic mice, streptozotocin-induced diabetic rats and type 2 diabetic rats [9, 18–23]. Phase II and III clinical trials with *L. speciosa* leaf decoctions showed promising results in the management of diabetes mellitus [24, 25]. Although it was thought that an insulin-like peptide hormone contributes to the hypoglycemic activity, it has been recently revealed that corosolic acid, a phytocomponent from *L. speciosa*, possesses free radical scavenging and hypoglycemic properties in animal model [24–27]. In addition the analgesic effect of root and fruit extracts (200 and 500 mg/kg body weight doses, respectively) was observed in animal model and the outcomes justify its traditional use [1, 28, 29]. The leaf extract also exhibited analgesic and anti-inflammatory activities against different chemical and thermal stimuli [30]. All these assays were found to be quite promising upon assays on animal model. Research should be conducted with flower extract of *L. speciosa* on animal model for the evaluation of different bioactivities since very limited number of studies have been conducted using the flowers of the plants.

To date, more than 40 compounds including triterpenes, tannins, ellagic acids, glycosides and flavones have been identified from the leaves of *L. speciosa*. Of the 14 compounds identified, four were new to the genus and family, and one was new to the species [31]. A series of ellagitannins and pentacyclic triterpenes, ellagic acid and its derivatives, a new tritrpenoid, corosolic acid, valoneic acid dilactone, quercetin, isoquercitin, virgatic acid, ursolic acid, β-sitosterol glucoside and some other phytocomponents were isolated and identified from *L. speciosa* extract [32–38].

We have been trying to investigate a few medicinal plants of Bangladesh for prominent biological activities [39-41]. The plants with promising history of traditional use are mainly chosen for research. The isolated phytoconstituents, traditional uses and the reported biological properties, have made *L. speciosa* a highly promising subject for further studies. The flowers of *L. speciosa* are very abundant in Bangladesh during the summer season and very limited number of research was carried out using the flowers of the plant. Therefore, we have conducted antioxidant, cytotoxic, thrombolytic, membrane stabilizing, antimicrobial, peripheral and central analgesic and hypoglycmic activity assays and phenobarbitone sodium–induced sleeping time test using *L. speciosa* flower extract.

## Methods

### Plant materials

In May 2015, *L. speciosa* flowers were procured from the premises of University of Dhaka, Bangladesh. This collection was identified by Mrs. Monira Begum. She is a botanist and PhD student of University of Dhaka. For this procurement, she provided a voucher specimen (DUSH - 5945) which was then preserved in Dhaka University Salar Khan Herbarium.

The plant materials were cleaned thoroughly and cut into small pieces. They were dried under sun and finally powdered when they were dry enough for proper grinding. Using 1.5 L Methanol, the powder (300 g) was kept macerated for a week under room temperature. Then the plant material was filtered and the corresponding filtrate was evaporated to dryness using a rotary evaporator at 40 °C and 50 r.p.m. to give the crude methanol extract. Following the modified Kupchan [42] partition protocol, the extract (5 g) was dissolved in 10% (*v*/v) aqueous methanol and transferred to a separating funnel. After pouring 100 ml hexane, the funnel was shaken and kept undisturbed so that the aqueous and organic layers could separate. The organic layer was collected. The process was repeated three times. 12.5 ml distilled water was added to the solution left after partitioning with hexane and then the mixture was extracted with 100 ml carbon tetrachloride. The organic layer was collected and the process was repeated three times. After that 16 ml distilled water was added to the funnel and the aqueous fraction was then extracted with 100 ml chloroform. The aqueous layer was partitioned with chloroform three times and the organic layer was collected. The aqueous methanolic fraction was further used as aqueous soluble fraction. All the partitionates were concentrated using rotary evaporator to yield hexane (HXSF, 1.0 g), carbon tetrachloride (CTCSF, 1.1 g), chloroform (CSF, 1.0 g) and aqueous (AQSF, 1.0 g) soluble fractions. They were then preserved in the refrigerator.

### Animal

For the assays on animal model, Swiss-albino mice were used. The mice were of either sex aging 5–6 weeks with an average weight of 25 g. The mice were provided by the International Centre for Diarrheal Diseases and Research, Bangladesh (ICDDR, B). The mice were healthy and free from any known infection. Before initiating the experiments, the animals were held and observed for 15 days so that they could adapt to laboratory circumstance. 120 × 30 × 30 cm cages were used and standard laboratory environment (room temperature 25 ± 2 °C; relative humidity 55–60%; 12 h light-dark cycle) was maintained. None was allowed inside the animal room during the dark phase. Experiments were conducted in the light cycle. The animals were fed with ICDDR, B formulated rodent food and tap water ad libitum. These were freely accessible to them. For the experiments the animals were picked randomly and were kept fasting overnight before experiments [43]. During experiment, any actual or considerable suffering or distress were monitored and lessened. For each test, the experimental unit was individual animal. After each experiment, carbon dioxide gas and cervical dislocation or decapitation method was used for anesthesia and euthanasia in order to minimize physical and mental sufferings of the animals. They were placed inside a transparent glass chamber so that they could be observed during exposure to carbon dioxide. Carbon dioxide gas cylinder, equipped with pressure regulator and flowmeter, was previously collected from local source. The gas was supplied in such a rate so that it could displace 10–30% of the chamber volume per minute. The gas concentration was slowly increased so that the animals lose consciousness because of loss of cerebral activity than suffocate to death due to exposure to 100% carbon dioxide. When all the animals became unconscious, the rate of gas flow was increased slowly to fill the chamber and decrease the time to death. After that, a secondary physical method (either decapitation or cervical dislocation) was utilized prior to disposal of the carcass. In decapitation, the animals were placed in the guillotine and their heads were chopped off. Since this process was untidy and uncomfortable for the operator, cervical dislocation method was used for euthanasia. In this method, a metal rod was placed quickly across the neck and pressed down hard while the body was jerked backwards by pulling on the tail. As a result, the neck became dislocated. The body was then examined for respiratory arrest and absence of heart beat before final disposal. Tests were conducted in accordance with The Swiss Academy of Medical Sciences and the Swiss Academy of Sciences formulated Ethical Principles and Guidelines for Scientific Experiments on Animals (1995) and performed under the approval of Ethics Committee of State University of Bangladesh through the submission of a research protocol (FHU/SUB/23/2014) before the study.

### Drugs and chemicals

*Streptokinase* and morphine were supplied by Beacon Pharmaceutical Ltd. and Gonoshastho Pharmaceuticals Ltd. Dhaka, Bangladesh, respectively. Diclofenac sodium BP and phenobarbitone sodium were obtained from Incepta Pharmaceuticals Ltd., Dhaka, Bangladesh. The rest of the drugs, reagents and solvents were obtained from Sigma-Aldrich, Munich, Germany.

#### Total phenolic content

Total phenolic content of the extract and the aqueous and organic soluble fractions was determined using Folin-Ciocalteau reagent [44]. At first, the reagent was diluted 10 times. 2.5 ml of the diluted reagent and 2.0 ml of sodium carbonate (7.5%, *w*/*v*) were added to the samples (2 mg/ml water). The sample was incubated for 15 min at 45 °C before taking the absorbance at 765 nm.

#### DPPH free radical scavenging activity assay

In this assay, the ability of the test samples to scavenge DPPH free radical was assessed using BHT and ascorbic acid as standards [45]. Calculated amount of different samples were measured and dissolved in methanol to get a concentration of 1000 μg/ml. Serial dilution of this solution gave different concentrations ranging from 500 to 0.977 μg /ml which were then mixed with 3.0 ml DPPH solution (20 μg/ml) in methanol. All the test tubes were then kept in dark for 30 min at room temperature before taking absorbance at 517 nm. The IC_50_ values (concentration of the extract providing 50% inhibition of oxidation) were calculated by plotting concentration of the samples versus percentage inhibition of free radical.

#### Brine shrimp lethality bioassay

In this method, *Artemia salina* leach (brine shrimp egg) was hatched in simulated sea water to give brine shrimp nauplii [46]. Dimethylsulfoxide (DMSO) was used for sample preparation and serial dilution was done to give different concentrations (400 μg/ml to 0.781 μg/ml). 5 ml simulated sea water was taken in pre-marked vials into which ten brine shrimp nauplii and test samples were transferred. The vials were then set aside for 24 h. After that, the vials were inspected using a magnifying glass and the number of survivors was counted. LC_50_ (median lethal concentration) values were calculated by plotting the logarithm of sample concentration against percentage mortality of the shrimp nauplii. The assay results were then compared with that of vincristine sulphate, which was used as positive control.

#### Thrombolytic activity assay

Following the method developed by Prasad et al. (2006) [47], the thrombolytic action of the plant extract and the aqueous and organic soluble fractions was determined. *Streptokinase* was used as positive control in this assay. Pre-weighed sterile micro-centrifuge tubes were taken. 5 ml venous blood was distributed in them. All the tubes were then incubated at 37 °C for 45 min. Serum was removed carefully after clot formation and the clot weight was determined. 100 μl aqueous solution of different samples (10 mg/ml water), 100 μl of *streptokinase* (positive control) and 100 μl of distilled water (negative control) were added separately to tubes containing pre-weighed clot. After incubation at 37 °C for 90 min, the released fluid was removed with care. Tubes were again weighed. The differences in weights were used to determine percentage of clot lysis according to the following equation:

$$ \mathrm{Percent}\ \mathrm{of}\ \mathrm{clot}\ \mathrm{lysis}=\left(\mathrm{weight}\ \mathrm{of}\ \mathrm{released}\ \mathrm{clot}/\mathrm{clot}\ \mathrm{weight}\right)\times 100\% $$

#### Membrane stabilizing activity

The ability of the test samples to inhibit heat induced haemolysis of human erythrocytes was assessed following the method of Omale et al. (2008) [48]. Samples (1 mg/ml methanol) were distributed between two duplicate sets of centrifuge tubes. 4.5 ml isotonic buffer was added to each of them to make the volume 5 ml. Another tube containing the isotonic buffer alone was used as negative control. 30 μl erythrocyte suspension was added to each tube and mixed gently by inversion. One set of the tubes was taken in water bath of 54 °C temperature and incubated for 20 min. The other set was placed in an ice bath of 0°-5 °C temperature. After centrifugation (3 min at 1300 g), the absorbance of the supernatant was determined at 540 nm using the isotonic buffer as blank. Percentage inhibition or acceleration of haemolysis was calculated using the following equation:

$$ \mathrm{Percent}\ \mathrm{inhibition}\ \mathrm{of}\ \mathrm{haemolysis}=100\ \mathrm{x}\ \left[1-\left({\mathrm{OD}}_2-{\mathrm{OD}}_1/{\mathrm{OD}}_3-{\mathrm{OD}}_1\right)\right]\% $$

where, OD_1_ = optical density of unheated test sample, OD_2_ = optical density of heated test sample and OD_3_ = optical density of heated control sample.

#### Antimicrobial screening

The extract and its different fractions were evaluated by disc diffusion method [49] to determine their antimicrobial potencies. The samples were taken at a dose of 400 μg/disc. In this investigation, ciprofloxacin and fluconazole (30 μg/disc) discs were used as antibacterial and antifungal references respectively. At first, the bacterial and fungal strains were transferred to previously sterilized and melted nutrient agar media. Then the preparations were poured onto sterilized petri dishes and allowed to solidify under laminar air flow hood. Then the petri dishes were kept in refrigerator until further use. Each test sample was dissolved in an appropriate solvent (chloroform or methanol) in such a way that 400 μg sample could be transferred to each disc. The discs were then dried with care under laminar air flow and placed on previously sterilized petri dishes with the help of semiautomatic pipette. Standard discs were placed as well. All the petri dishes were placed inside refrigerator at 4 °C for 24 h and then inside an incubator at 37 °C for 24 h. After incubation, zone of inhibition was measured. The Institute of Nutrition and Food Science (INFS) of University of Dhaka supplied the pure microbial cultures.

#### Peripheral analgesic activity

In peripheral analgesic activity assay, acetic acid was injected intraperitonially to induce abdominal pain manifested by writhing in mice [50]. The samples were evaluated by determining their ability to inhibit writhing.

Each group consisted of five mice. Distilled water (10 ml/kg) and diclofenac sodium (50 mg/kg body weight) were given orally to the mice of negative and positive control groups, respectively. The crude methanol extract (200 and 400 mg/kg doses) was administered orally to mice of other two groups. After 40 min, 1% volume/volume acetic acid (10 ml/kg dose) was injected intraperitonially. After giving a resting period of 10 min, the number of writhing given by each animal was counted for the next 10 min. The percent inhibition of pain induced by acetic acid was calculated according to the following formula:

$$ \mathrm{Percentage}\ \mathrm{inhibition}=\frac{\left[\mathrm{Mean}\ \mathrm{number}\ \mathrm{of}\ \mathrm{writhing}\ \left(\mathrm{control}\right)-\mathrm{Mean}\ \mathrm{number}\ \mathrm{of}\ \mathrm{writhing}\ \left(\mathrm{test}\right)\right]\times 100\%}{\mathrm{Mean}\ \mathrm{number}\ \mathrm{of}\ \mathrm{writhing}\ \left(\mathrm{control}\right)} $$

#### Central analgesic activity

Tail flicking method was used to determine central analgesic activity [51]. Before administering anything, the tip of the tail (last 1–2 cm) of all mice was immersed on the radiant heat source. The tail flicking response was considered as the end point. For preventing damage to the tail, a cut off period of 15 s was set. Here, a total of twenty mice divided into four groups were used. Mice of negative control group were given 1% Tween-80 in saline mixture (0.1 ml/10 mg) orally while mice of positive control group received morphine (2 mg/kg) subcutaneously. The crude methanol extract (200 and 400 mg/kg doses) was administered orally to mice of other two groups. The tail flicking response was then recorded after 30 min, 60 min and 90 min of administration of samples. The pain inhibition percentage (PIP) was calculated according to the following formula:

$$ \mathrm{Pain}\ \mathrm{inhibition}\ \mathrm{percentage}\ \left(\mathrm{PIP}\right)=\left(\left({\mathrm{T}}_1-{\mathrm{T}}_0\right)/{\mathrm{T}}_0\right)\ \mathrm{x}\ 100 $$

where, T_1_ is post-drug latency and T_0_ is pre-drug latency.

#### Hypoglycemic activity assay

Tail tipping method was used to determine hypoglycemic effect of the test samples in mice [52]. At first, blood was withdrawn from the tip of the tail and the sugar level was measured by using glucometer. Here, a total of twenty mice divided into four groups were used. The mice of negative and positive control groups received 1% Tween-80 in saline mixture (0.1 ml/10 mg) and Glibenclamide (5 mg/kg), respectively. The methanolic crude extract (200 mg/kg and 400 mg/kg doses) was given orally to mice of treatment groups. After an hour, 10% glucose solution (2 g/kg body weight) was given orally to all the mice. The blood sugar level was again recorded after 1^st^, 2^nd^ and 3^rd^ hour of administration of glucose solution.

#### Phenobarbitone sodium-induced sleeping time

In phenobarbitone sodium-induced sleeping time test [53], fifteen mice were equally divided into three groups. The treatment groups received the crude methanol extract (200 and 400 mg/kg body weight doses) while the control group received saline water containing Tween-80 solution alone. After 30 min, all the animals received phenobarbitone sodium at a dose of 25 mg/kg body weight intraperitonially. Phenobarbitone sodium was given to induce sleep. All the mice were then monitored to observe the time of onset of sleep and total sleeping time.

### Statistical analysis:

Three replicates of each sample were used for statistical analysis and all of the values are expressed as the mean ± standard deviation (SD). The results were evaluated by a two-tailed non-parametric pair t-test. *P* < 0.05 was considered statistically significant.

## Results

Our research attempted to assess the crude methanol extract of *L. speciosa* flowers for different bioactivities by mice and non-mice based assays.

In antioxidant activity assay, the highest free radical scavenging activity was demonstrated by the chloroform soluble fraction of *L. speciosa* extract (IC_50_ = 4.20 ± 0.41 μg/ml). On the other hand, while evaluating the test samples for cytotoxic potencies, the hexane soluble fraction exhibited the lowest LC_50_ value (LC_50_ = 2.00 ± 0.31 μg/ml) compared to other samples (Table 1).

**Table 1:** Antioxidant and cytotoxic activities of *L. speciosa* flower extract and soluble fractions

Samples/ Standards	Total phenolic content (mg of GAE/ g of dried extract)	Free radical scavenging activity IC_50_ (μg/ml)	Brine shrimp lethality bioassay LC_50_ (μg/ml)
ME	17.71 ± 0.32	4.50 ± 0.11	25.70 ± 0.19
HXSF	11.15 ± 0.56	32.11 ± 0.58	2.00 ± 0.31
CTCSF	9.94 ± 0.13	5.15 ± 0.21	15.06 ± 0.43
CSF	1.42 ± 0.44	4.20 ± 0.41	18.37 ± 0.32
AQSF	19.34 ± 0.49	10.92 ± 0.33	84.02 ± 0.13
Vincristine sulfate	–	–	0.45 ± 0.04
BHT	–	27.50 ± 0.54	–
Ascorbic acid	–	5.80 ± 0.21	–

ME = Methanolic crude extract; HXSF = Hexane soluble fraction; CTCSF = Carbon tetrachloride soluble fraction; CSF = Chloroform soluble fraction; AQSF = Aqueous soluble fraction; BHT = Butylated hydroxytoluene; GAE = Gallic acid equivalent

Among the test samples of *L. speciosa*, the carbon tetrachloride soluble fraction showed the highest capacity to promote lysis of clot (64.80 ± 0.27% of clot lysis) as well as to inhibit haemolysis of RBCs under heat-induced condition (41.90 ± 0.10%). Both the findings were comparable to the standards used in the respective assays (Table 2).

**Table 2:** Thrombolytic and membrane stabilizing activities of *L. speciosa* flower extract and soluble fractions

Samples/ Standard	% of lysis of RBCs	% Inhibition of haemolysis induced by heat
ME	38.43 ± 0.24	32.62 ± 0.18
HXSF	47.20 ± 0.49	28.56 ± 0.12
CTCSF	64.80 ± 0.27	41.90 ± 0.10
CSF	46.91 ± 0.62	40.81 ± 0.25
AQSF	58.63 ± 0.39	16.03 ± 0.37
Water	3.79 ± 0.55	–
*Streptokinase*	66.77 ± 0.36	–
Acetyl salicylic acid	–	42.12 ± 0.38

ME = Methanolic crude extract; HXSF = Hexane soluble fraction; CTCSF = Carbon tetrachloride soluble fraction; CSF = Chloroform soluble fraction; AQSF = Aqueous soluble fraction

In disc diffusion assay, all the test samples were evaluated by zones of inhibition which were formed by inhibiting the growth of different microorganisms. A 19.0 mm zone of inhibition was observed against *Staphylococcus aureus*. It was demonstrated by the carbon tetrachloride soluble fraction. Another 16.0 mm zone of inhibition was measured against *Bacillus megaterium* demonstrated by the crude methanol extract (Table 3).

**Table 3:** Antimicrobial activity of test samples of *L. speciosa*

Test microorganisms	Diameter of zone of inhibition (mm)					
	ME	HXSF	CTCSF	CSF	AQSF	Ciprofloxacin/ Fluconazole (30 μg/disc)
Gram positive bacteria						
*Bacillus cereus*	8.0 ± 0.12	–	–	7.00 ± 0.92	–	45 ± 2.01
*Bacillus megaterium*	16.0 ± 0.43	15.0 ± 0.73	–	–	–	42 ± 1.17
*Bacillus subtilis*	9.0 ± 0.56	–	9.0 ± 0.33	–	–	42 ± 0.73
*Staphylococcus aureus*	10.0 ± 0.57	–	19.0 ± 0.48	7.0 ± 1.45	7.0 ± 1.3	42 ± 0.23
*Micrococcus luteus*	9.0 ± 0.16	–	6.0 ± 0.45	7.0 ± 1.11	7.0 ± 0.92	42 ± 0.56
Gram negative bacteria						
*Escherichia coli*	8.0 ± 0.54	–	7.0 ± 0.81	7.0 ± 0.45	–	42 ± 0.43
*Pseudomonas aeruginosa*	10.0 ± 0.36	–	7.0 ± 0.32	8.0 ± 0.31	–	42 ± 1.11
*Salmonella paratyphi*	8.0 ± 0.68	–	–	10.0 ± 0.55	8.0 ± 1.56	47 ± 2.33
*Salmonella typhi*	–	–	9.0 ± 0.17	–	9.0 ± 0.41	45 ± 0.73
*Shigella boydii*	7.0 ± 0.87	–	6.0 ± 0.66	7.0 ± 1.90	–	34 ± 0.58
*Shigella dysenteriae*	10.0 ± 0.73	–	9.0 ± 0.25	12.0 ± 0.22	7.0 ± 0.83	42 ± 0.22
*Vibrio mimicus*	6.0 ± 0.21	–	6.0 ± 0.16	8.0 ± 0.48	–	40 ± 0.45
*Vibrio parahemolyticus*	9.0 ± 1.56	–	6.0 ± 1.12	8.0 ± 2.12	7.0 ± 0.21	35 ± 0.44
Fungi						
*Saccharomyces cerevisiae*	7.0 ± 1.12	–	7.0 ± 0.53	7.0 ± 1.43	6.0 ± 0.21	38 ± 0.11
*Aspergillus niger*	8.0 ± 0.43	–	8.0 ± 0.48	7.0 ± 0.33	5.0 ± 0.54	37 ± 0.33

ME = Methanolic crude extract; HXSF = Hexane soluble fraction; CTCSF = Carbon tetrachloride soluble fraction; CSF = Chloroform soluble fraction; AQSF = Aqueous soluble fraction

*L. speciosa* flower extract was found to exhibit dose dependent inhibition of acetic acid induced writhing in Swiss albino mice. The outcome of peripheral analgesic activity assay was not found to be significant compared to the standard diclofenac sodium (Table 4).

**Table 4:** Effect of *L. speciosa* flower extract on acetic acid-induced writhing in mice

Groups	Number of writhing	Inhibition of writhing (%)
Control (10 ml/kg)	19.6	
Diclofenac sodium (50 mg/kg)	5.00 ± 0.68	74.49
Methanol extract (200 mg/kg)	19.41 ± 0.26	0.97
Methanol extract (400 mg/kg)	16.33 ± 0.12	16.68

Values are expressed as Mean ± SD from the experiments

*P < 0.05; **P < 0.01 vs. control; *n* = 5

In central analgesic activity assay by tail-flick method, 48.24% elongation of the reaction time was observed after 30 min of administration of the crude methanol extract (400 mg/kg body weight) of the flowers of *L. speciosa*. (Tables 5 and 6).

**Table 5:** Effect of *L. speciosa* flower extract on tail flicking time of mice

Groups	Latency period			
	0 min	30 min	60 min	90 min
Control	6.03 ± 0.47	6.26 ± 0.57	6.16 ± 0.89	6.23 ± 0.47
Morphine (2 mg/kg)	10.80 ± 0.91	16.5 ± 1.03	15.96 ± 1.17	16.16 ± 1.07
Methanol extract (200 mg/kg)	6.80 ± 0.54	4.25 ± 0.22	7.40 ± 0.86	5.41 ± 0.75
Methanol extract (400 mg/kg)	6.44 ± 0.33	3.24 ± 0.43	5.70 ± 0.61	4.22 ± 0.79

**Table 6:** Anti-nociceptive activity of methanolic crude extract of *L. speciosa* flower

Groups	% Pain inhibition		
	30 min	60 min	90 min
Morphine (2 mg/kg)	163.58 ± 2.38	159.09 ± 2.03	159.39 ± 1.89
Methanol extract (200 mg/kg)	32.11 ± 0.44	20.13 ± 1.03	13.16 ± 0.72
Methanol extract (400 mg/kg)	48.24 ± 0.91	7.47 ± 0.27	32.26 ± 0.41

Values are expressed as Mean ± SD from the experiments

*L. speciosa* flower extract was found to lower the blood sugar level significantly while evaluating its hypoglycemic activity by tail tipping method. Its efficacy was evident even after 3rd hour of administration of the glucose solution. The blood sugar level was reduced by 48.85 and 56.12% at 200 and 400 mg/ kg body weight doses, respectively (Table 7 and Table 8).

**Table 7:** Effect of *L. speciosa* flower extract on blood glucose of Swiss albino mice

Groups	Blood Glucose Level (mmol/L)			
	0 min	1^st^ hour	2^nd^ hour	3^rd^ hour
Control Group	5.98 ± 0.12	5.72 ± 0.49	5.70 ± 0.34	5.63 ± 0.56
Standard Group (5 mg/kg)	5.96 ± 0.31	2.00 ± 0.07	2.54 ± 0.13	2.76 ± 0.16
Methanol extract (200 mg/kg)	4.66 ± 0.23	5.22 ± 0.37	3.04 ± 0.11*	2.88 ± 0.33**
Methanol extract (400 mg/kg)	4.64 ± 0.41	2.88 ± 0.83**	2.60 ± 0.47**	2.47 ± 0.66**

Values are expressed as mean ± SD; * *P* < 0.05; **P < 0.01 vs. control; *n* = 5

**Table 8:** Percentage of blood glucose level reduction in Swiss albino mice

Groups	% of blood glucose level reduction		
	1^st^ hour	2^nd^ hour	3^rd^ hour
Control Group	4.35	4.68	5.85
Standard Group (5 mg/kg)	65.03	55.43	50.98
Methanol extract (200 mg/kg)	8.74	46.67	48.85
Methanol extract (400 mg/kg)	49.65	54.39	56.12

In phenobarbitone sodium–induced sleeping time test, *L. speciosa* flower extract delayed the onset of sleep (30.22 min and 38.61 min at 200 and 400 mg/kg body weight doses, respectively) compared to the control group (15.8 min). The extract was also found to shorten total sleeping time (99.45 min and 107.6 min at 200 and 400 mg/kg body weight doses, respectively) compared to the control group (118.6 min) (Table 9).

**Table 9:** Effect of *L. speciosa* flower extract on phenobarbitone sodium–induced sleep

Groups	Time of onset of sleep (minutes)	Total sleeping time (minutes)
Control (10 ml/kg)	15.8 ± 1.19	118.6 ± 2.81
Methanol extract (200 mg/kg)	30.22 ± 2.20	99.45 ± 2.85
Methanol extract (400 mg/kg)	38.61 ± 1.76	107.6 ± 3.21

Values are expressed as Mean ± SD from the experiments

## Discussion

The free radical scavenging and cytotoxic activities of *L. speciosa* test samples could be attributed to the high phenolic and flavonoid content in the leaves and flower petals and also to some phytocomponents like corosolic acid, ellagic acid, gallic acid and quercetin that have been found to be present in *L. speciosa*. These compounds are well known for their antioxidant potencies [54–58]. Again quercetin has been reported to have cytotoxic potential from its ability to increase the ROS production [59]. Treatment of human prostate cancer was reported to be successful with gallic acid [60].

The observations in thrombolytic activity assay may have revolutionary significance since it may lead to the discovery of new cardiovascular drugs [1]. Blood clot formation is a complex process that involves series of events [61]. The ability of the test samples to promote thrombolysis may pave the way of discovering novel thrombolytic agents from *L. speciosa* flower extract. In addition, according to ayurvedic medicine, arjunolic acid can prevent necrosis of the myocardium and clot formation [62]. This phytoconstituent has already been extracted from *L. speciosa* and its presence can be correlated with the extract’s thrombolytic potential.

*L. speciosa* extract and its aqueous and organic soluble fractions reduced heat-induced haemolysis of RBC. Membranes of lysosome and red blood cells have similar structural features [63]. Inflammation occurs due to leakage of serum protein and fluids into tissues. By decreasing membrane permeability and subsequently preventing drainage of proteins and fluids, membrane stability can be ensured and anti-inflammatory response can be manifested [64]. Arjunolic acid has been proved to have the ability to affect *cyclooxygenase* catalyzed arachidonic acid metabolism. This phytochemical might have contributed to the plant’s anti-inflammatory activity [62, 65]. On the other hand, quercetin and isoquercitrin were found to be effective eosinophilic inflammation suppressors. These phytocomponents can be used for treating allergies [66]. Again, gallic acid was reported to exhibited anti-inflammatory activity in animal model [67]. All these phytochemicals reported from *L. speciosa,* might have contributed to its membrane stabilizing potential.

Test samples from *L. speciosa* flowers were observed to give zones of inhibition against different microorganisms due to the presence of well known plant metabolites [68]. The leaf and seed extracts of *L. speciosa* showed prominent antimicrobial activity against different micro-organisms [69, 70]. Arjunolic acid and Asiatic acid, reported to be isolated from *L. speciosa*, have been proved to possess antimicrobial potencies [62, 71].

The hypoglycemic activity of *L. speciosa* extract may be due to the presence of corosolic acid in the plant. This phytocomponent not only increases insulin secretion [17, 72] but also stimulates cellular glucose uptake [73]. In short, corosolic acid has insulin-like effect [73–75]. Like corosolic acid, three active ellagitannins: lagerstroemin, flosin B and reginin A, reported from *L. speciosa* extracts, also promote glucose uptake in fat cells [15, 76–79]. On the other hand, corosolic acid has been found to have both free radical scavenging and hypoglycemic properties in animal model. It has been suggested that ROS destroys β-cells of pancreas [25]. Thus by scavenging free radicals, corosolic acid is indirectly keeping β-cells of pancreas functional thereby demonstrating hypoglycemic effect [9, 22, 80]. However, recently a glucose uptake assay has indicated that not corosolic acid but gallotannins can be accounted for the hypoglycemic activity. Gallotannins are phytocomponents already reported from the species under investigation. Among the gallotannins, penta-O-galloyl-glucopyranose stimulates transportation of glucose into the cell to a greater extent than lagerstroemin [81]. Another phytocomponent reported from the plant is arjunolic acid which was found to be beneficial against both type I and type II diabetes [62].

Arjunolic acid might be credited for the analgesic activity of *L. speciosa* extract since this phytocomponent has been reported to have influences on COX pathway [62].

The outcome of the assay in which sleep was induced using phenobarbitone sodium, is a major finding and need further investigation for phytochemicals responsible for the effect.

## Conclusion

Although *L. speciosa* is an ornamental plant and planted as a road side tree in Bangladesh, the plant has long history of folkloric uses. Investigation on different plant parts has led to the isolation of active phytochemicals and understanding of medicinal benefits of the plant that can be correlated to its traditional uses. From our study, we can come to the conclusion that like other plant parts, the flowers of the plant have major bioactivities. *L. speciosa* samples demonstrated noteworthy potentials in scavenging free radicals, promoting clot lysis and stabilizing RBC membrane under heat induced condition. Analgesic activity was not found to be that prominent but the outcome of hypoglycemic activity assay justifies its traditional use in diabetes. Recent objective of the global pharmaceutical industries is to develop novel drugs of plant origin on the basis of the history of traditional uses of those plants. The phytoconstituents that are actually responsible for the observed bioactivities, should be isolated by different experimental methods, identified and finally can be involved in biological activity assays. Thus, it will be possible to understand their mechanism of action. Therefore, in the conclusion, it can be said that *L. speciosa* flowers are excellent candidate for future investigation for potent phytocomponents with biological activities.

## Abbreviations

BHT: Butylated hydroxytoluene
CNS: Central nervous system
COX: *Cyclooxygenase*
DMSO: dimethylsulfoxide
DPPH: 1, 1-diphenyl-2-picrylhydrazyl
GAE: Gallic acid equivalent
ICDDR, B: International Centre for Diarrheal Diseases and Research, Bangladesh
RBCs: Red blood cells
ROS: Reactive Oxygen Species
